# “I Haven’t Had Moose Meat in a Long Time”: Exploring Urban Indigenous Perspectives on Traditional Foods in Saskatchewan

**DOI:** 10.3390/nu16152432

**Published:** 2024-07-26

**Authors:** Mojtaba Shafiee, Samer Al-Bazz, Michael Szafron, Ginny Lane, Hassan Vatanparast

**Affiliations:** 1 College of Pharmacy and Nutrition, University of Saskatchewan, Saskatoon, SK S7N 5A9, Canada; mojtaba.shafiee@usask.ca (M.S.); saa331@mail.usask.ca (S.A.-B.); 2 School of Public Health, University of Saskatchewan, Saskatoon, SK S7N 2Z4, Canada; michael.szafron@usask.ca; 3 Margaret Ritchie School of Family and Consumer Sciences, University of Idaho, Moscow, ID 83843, USA; vlane@uidaho.edu

**Keywords:** indigenous people, urban setting, traditional foods, nutritional benefits, barriers, Canada

## Abstract

This qualitative study investigates the perspectives of urban Indigenous individuals in Saskatchewan, Canada, regarding their consumption of traditional foods. Through in-depth, semi-structured interviews with 14 participants across Saskatoon, Regina, and Prince Albert, the research aimed to uncover the benefits, risks, and barriers associated with acquiring and consuming traditional foods. Participants emphasized the nutritional advantages of traditional foods, such as higher nutrient density and absence of industrial additives, which they linked to improved health outcomes and alignment with Indigenous biology. The study also highlighted the vital role of traditional foods in maintaining cultural identity and fostering community connections through practices of food sharing and intergenerational knowledge transfer. However, significant challenges were identified, including economic and physical barriers to access, environmental degradation, and regulatory issues that restrict the availability of traditional foods in urban settings. The findings suggest a complex landscape where cultural practices are both preserved and challenged within the urban environment. This study contributes to the broader understanding of how Indigenous populations navigate the preservation of their culinary heritage in the face of modern economic and environmental pressures, providing insights for policy and community-based interventions aimed at supporting Indigenous food sovereignty.

## 1. Introduction

For generations, Indigenous peoples in Canada have utilized their profound knowledge of the environment and traditional food systems to sustain themselves from the land [1]. These traditional food systems, critical to their livelihood, subsistence lifestyle, and overall health and well-being, encompass much more than just sustenance [1]. As described by Kuhnlein and Chan [2], the traditional food system “includes all of the food species that are available to a particular culture from local natural resources and the accepted patterns for their use within that culture”. This definition extends to the sociocultural significance of these foods, encompassing their acquisition, processing, chemical composition, and the roles they play across different ages and genders within a culture. It also considers the nutritional and health implications for those who consume these foods [2]. Traditional foods, often referred to as country foods, predominantly consist of animal and plant species harvested from the natural environment, including wild meats, various fish and bird species, plants, and berries [1]. Indigenous peoples acquire these foods through traditional activities such as hunting, fishing, and gathering, which vary with the seasons. These practices do more than provide food; they promote physical health, uphold strong cultural identities and values, and deepen the connection to the land and traditional ways of life [1].

Research indicates that traditional foods are often more nutrient-dense than conventional modern diets that are typically rich in carbohydrates, particularly refined sugars, saturated fats, and sodium [3,4,5]. The presence of traditional foods even in small amounts in the diet of Indigenous peoples often results in a higher diet quality [6,7,8]. However, the impact of colonial assimilation policies has triggered an “accelerated nutrition transition”, a phenomenon that describes a rapid shift towards a less healthy diet resulting from decreased reliance on traditional foods and increased reliance on market-based foods [9,10]. The decline in traditional food consumption and over-reliance on market foods is linked to rising rates of obesity and nutrition-related chronic diseases, such as type 2 diabetes and cardiovascular disease among Indigenous populations [11,12,13].

Previous research highlights that the utilization of traditional foods by Indigenous Peoples in Canada, particularly those living on reserves, is limited by a variety of factors [14,15,16,17]. These include financial and household constraints, climate change, government regulations, lack of time, fear of contamination, and absence of a hunter in the household [14,15,16,17]. Historically, the focus of research on traditional foods has predominantly centered on Indigenous communities living on reserves. However, with around 60% of the Indigenous population in Canada now residing in urban areas [18], it is essential to deepen our understanding of the role traditional foods play within urban contexts. The 2021 Census revealed that the Indigenous population surpassed 1.8 million, making up 5% of Canada’s overall population and marking a 9.4% rise since 2016 [18]. Furthermore, a notable shift toward urban living has been observed, with 1,090,240 Indigenous people now residing in urban areas—a growth of 11.5% from 2016 [18]. Saskatchewan figures prominently in this demographic shift, hosting 10.4% of Canada’s Indigenous population [19]. Urban centers such as Saskatoon, Regina, and Prince Albert are increasingly important to this demographic, with Indigenous residents making up 15.9% (29,880 individuals) in Saskatoon, 12.4% (23,285 individuals) in Regina, and 8.6% (16,125 individuals) in Prince Albert [19]. The relocation of Indigenous peoples to these cities is often motivated by better opportunities for education, employment, healthcare, and other urban amenities [20,21]. Yet, this urban migration also introduces distinct challenges, especially in maintaining cultural continuity, which encompasses traditional food practices [22]. Urban Indigenous communities face numerous systemic barriers, including socioeconomic disparities and racial discrimination [23], which can hinder the preservation of their cultural heritage. Despite these challenges, there is a resilient effort among these communities to preserve and adapt their traditional food practices within urban environments. This endeavor helps bridge the gap between urban living and cultural preservation and plays a crucial role in sustaining community identity and health in urban settings.

This study aims to explore the perceptions, benefits, facilitators, and challenges associated with the consumption of traditional foods among urban Indigenous peoples in Saskatchewan, Canada, with a focus on three primary urban centers: Saskatoon, Regina, and Prince Albert. Given the critical role of traditional foods in the health and food security of Indigenous populations, and considering their increasing presence in urban settings, this research seeks to provide insights into how traditional foods are integrated into the lives of urban Indigenous residents, the values associated with these foods, and the obstacles to their consumption. By capturing the Indigenous peoples’ voices, the study aims to highlight actionable pathways that can support the revitalization and integration of traditional dietary practices in urban environments.

## 2. Materials and Methods

### 2.1. Study Design

This research was carried out using a qualitative approach, specifically through inductive thematic analysis [24]. Our choice of an inductive method facilitated the emergence of themes and patterns from the data, allowing for analysis that was not constrained by preconceived hypotheses [25]. Such an approach helps to ensure that our conclusions are closely derived from the participants’ own experiences and viewpoints. To strengthen our analysis through an intersectionality lens, we employed the Social Ecological Model (SEM) as a theoretical framework [26]. This approach enabled us to explore the various levels of influence on traditional food practices, from individual factors to broader societal and policy influences. This study is reported in accordance with the Consolidated Criteria for Reporting Qualitative Research (COREQ) checklist [27]. The Behavioural Research Ethics Board (Beh-REB) at the University of Saskatchewan granted ethical approval for this research (Approval ID: Beh 3620).

### 2.2. Participant Recruitment and Sampling Strategies

The research was conducted across three key urban centers in Saskatchewan, Canada—Saskatoon, Regina, and Prince Albert—during September and October 2023. Participants were primarily recruited through a partnership with the Saskatchewan Network Environments for Indigenous Health Research (SK-NEIHR) and the Saskatoon Food Bank and Learning Centre. Recruitment efforts included the distribution of a detailed poster and an email message, which were also shared via SK-NEIHR’s social media platforms. Additionally, physical posters were displayed at the Saskatoon Food Bank and Learning Centre to encourage interested individuals to contact the research team via the provided email address. Our study included participants who were 18 years of age or older, identified themselves as Indigenous, and lived in one of the three cities mentioned.

We used a purposive sampling method to concentrate on Indigenous health perspectives within these urban settings. This strategy allowed us to selectively target individuals who fulfilled our study’s inclusion criteria, ensuring that our participants’ characteristics were closely aligned with our research aims. Alongside purposive sampling, we employed snowball sampling techniques. This method leveraged our initial participants’ networks, encouraging them to inform peers about the study, thus broadening our reach and facilitating the inclusion of participants who might otherwise have been unreachable through our direct recruitment efforts alone.

### 2.3. Data Collection

For our study, we employed a qualitative methodology, using semi-structured interviews to explore the experiences and viewpoints of urban Indigenous adults. These interviews were conducted through Zoom, an online video conferencing tool, to enhance accessibility and convenience for the participants. Additionally, we provided an option for face-to-face interviews to accommodate those preferring a more direct interaction; however, no participants requested a face-to-face interview.

The interviews were facilitated by a single researcher, M.Sh., a male PhD candidate with expertise in food security and Indigenous health. As an immigrant to Canada and a member of an ethnic minority, M.Sh. brought a unique perspective that enhanced his understanding and sensitivity to the participants’ experiences. There was no pre-existing relationship between M.Sh. and the participants, which helped minimize bias during data collection. M.Sh. engaged in reflective practices throughout the research process to further address the potential impact of his social standing and outsider status. He worked to maintain an open and respectful communication environment, carefully listening to participants’ stories and perspectives without imposing his views.

The interview scheduling was driven by the participants selecting their preferred date and time, ensuring that their convenience and availability were prioritized. Preceding the interviews, participants were thoroughly briefed on the research team and the objectives of the study. Consent forms were sent out via email at least one day before their scheduled interview. Each participant was interviewed once, with no follow-up sessions. At the start of each interview, M.Sh. introduced himself, outlined his academic and professional background, and clarified the study’s purpose.

The semi-structured nature of the interviews allowed for a dynamic exchange, providing the flexibility to explore emergent topics while ensuring all guiding questions were addressed. This format was instrumental in gaining a comprehensive understanding of the participants’ perspectives and experiences. The interviews were structured around key questions that guided the discussions:

1. What do you think of when you hear the term “traditional foods”?
2. Can you identify any benefits of consuming traditional foods?
3. Do you perceive any risks associated with consuming traditional foods?
4. Are there reasons you might avoid or limit your consumption of traditional foods?

Interviews continued until data saturation was achieved, indicating that no new information was emerging from the discussions [28]. All data were securely maintained in a password-protected folder within the University of Saskatchewan’s Datastore system.

### 2.4. Data Analysis

The data collection involved audio-recording the interviews via Zoom, with each session being transcribed verbatim by the interviewer, M.Sh., to capture the data accurately for analysis. Alongside transcription, M.Sh. also made detailed notes during the interviews to aid in subsequent analysis. To ensure the reliability of data interpretation, a member-checking process was implemented; the transcribed interviews were shared with the participants for confirmation. This process allowed participants to review the accuracy of the transcripts and ensure that their perspectives were correctly represented [29], with any suggested revisions to be returned within a week.

The analysis was performed using inductive thematic analysis. Initially, M.Sh., the first author, conducted open coding by identifying transcript phrases related to the research questions. The coding scheme was then reviewed and refined in collaboration with the second author, S.A., with any disagreements resolved through re-coding. This iterative process led to the development of sub-themes. Further refinement and validation of these sub-themes involved discussions with H.V., the last author. The final themes were determined by combining these refined sub-themes. Sub-themes that were related and discussed similar ideas were merged to form broader themes. However, there were instances where certain sub-themes were straightforward and distinct in their content and implications. These sub-themes, due to their unique and self-contained nature, were thus considered and presented as main themes.

Throughout the analysis, NVivo version 12 was utilized, facilitating a systematic and organized review of the data. This software helped in identifying and categorizing key themes and insights pertinent to the study’s aims. Additionally, a word cloud was generated using www.wordart.com to visualize the most frequently mentioned terms related to the perception of traditional foods, as well as the benefits and risks associated with them, as discussed by the participants.

## 3. Results

This research gathered perspectives from 14 urban Indigenous individuals residing in three Saskatchewan cities (i.e., Saskatoon, Regina, and Prince Albert). Participant demographics and characteristics are detailed in Table 1. In terms of Indigenous identity, 12 participants identified as First Nation, one as Métis, and one did not specify their Indigenous identity. For their place of residence, seven lived in Saskatoon, five in Regina, and two in Prince Albert. Gender distribution among the participants showed that ten were female and four were male. Regarding educational background, eight participants had an undergraduate education, three had completed high school, one had a graduate education, and the remainder (one each) had non-degree or professional certifications. Employment status revealed that eight of the participants were employed while six were unemployed. The household sizes varied, with four living in two-person households, three in four-person households, and three in households of five or more. In terms of housing, eleven participants were renting, two owned their homes, and one did not specify their housing situation. The average age of participants was 39.8 years, with a standard deviation of 11.7 years, ranging from 21 to 61 years old. The annual household income averaged $72,600, with a standard deviation of $30,692, spanning from $20,000 to $120,000.

The duration of the interviews ranged from 20 to 49 min, with an average time of 29 min per interview. Data saturation was attained after the twelfth interview. To ensure completeness, two further interviews were carried out, which confirmed that data saturation had been reached, as these sessions did not introduce any new insights.

### 3.1. Perception of Traditional Foods

The perception and definition of traditional foods among urban Indigenous peoples in Saskatchewan encompass a profound connection to cultural heritage, environmental sustainability, and traditional practices. The following themes highlight how participants define and perceive traditional foods, reflecting their cultural significance, reliance on the natural environment, and traditional methods of procurement.

#### 3.1.1. Theme 1: Foods with Ancestral Heritage and Cultural Meaning

Participants often defined traditional foods as those consumed by their ancestors, highlighting their deep cultural and historical significance. This theme encompasses not only the types of foods eaten but also the traditional ways of life that accompany their preparation and consumption. Foods such as Bannock, pemmican, and various meats commonly featured in traditional feasts and gatherings, serving as a direct link to past generations and a means of preserving cultural identity and heritage.

*“When I hear the word traditional foods, what comes to mind is definitely foods that our ancestors used to eat, the traditional ways of life and having feasts and the traditional foods that my grandparents and great-grandparents used to eat; so a lot of Bannock, pemmican, moose meat, etc.”*—Participant 2, Female

#### 3.1.2. Theme 2: Locally Sourced and Land-Based Foods

This theme emphasizes the importance of consuming foods that are native to the land, highlighting a diet based on locally sourced, natural ingredients. It reflects a lifestyle of sustainability and respect for the environment, where food sources change with the seasons and availability.

*“Traditional food for us means just eating off the land as much as we can so we eat a lot of moose. It just changes from season to season. In the summer we eat a lot of fish.”*—Participant 4, Female

#### 3.1.3. Theme 3: Food Acquired through Traditional Methods

A significant aspect of traditional foods involves the methods of obtaining them—primarily through fishing, hunting, and gathering. This theme reflects a practical approach to food sourcing that is deeply embedded in traditional practices. Participants detailed how they actively hunt and gather food like wild meat and berries, practices that not only provide physical nourishment but also teach valuable skills and ensure the transmission of knowledge from one generation to the next.

*“I think of wild berries and wild meat and things that have been hunted and gathered.”*—Participant 5, Female

**Figure 1:**
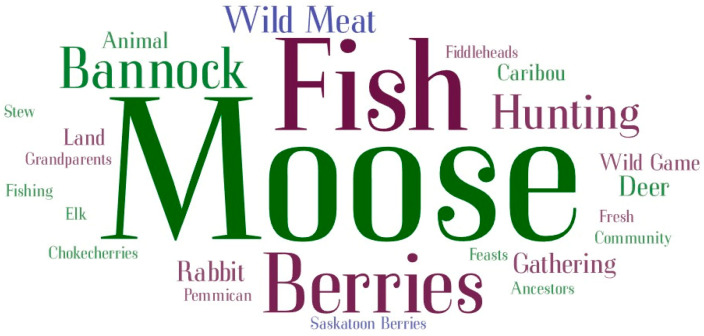
Word cloud of frequently used words in interviews on perception of traditional foods.

The word cloud for the most frequently used words in the interviews on perception of traditional foods is shown in Figure 1.

### 3.2. Benefits of Traditional Foods

In exploring the benefits of traditional foods among Indigenous populations, six key themes were revealed: (1) Health and Nutritional Benefits; (2) Cultural and Community Connection; (3) Physical and Mental Well-being; (4) Economic and Cost Benefits; (5) Environmental and Ethical Considerations; and (6) Personal Preferences and Experiences (Table 2).

#### 3.2.1. Theme 1: Health and Nutritional Benefits

This theme focuses on the numerous health and nutritional benefits associated with traditional foods. Participants shared insights on various aspects, including nutrient density, lack of industrial processing, medicinal properties, and their alignment with Indigenous biology.

##### Nutrient Density and Quality

Traditional foods, such as moose meat, berries, and fish, are praised for their high nutrient content and lean protein, offering significant health benefits:

*“Moose meat, for example, … moose meat is like almost zero fat and high in protein, which is extremely healthy for you.”*—Participant 1, Female

##### Lack of Industrial Processing and Additives

The absence of industrial processing and additives in traditional foods is cited as a key factor in their healthfulness. These foods are considered superior to store-bought alternatives that may contain hormones, preservatives, and other unhealthy ingredients:

*“And then there’s health benefits like you know that these animals aren’t getting pumped full of hormones, and there isn’t ingredients that you can’t pronounce in them.”*—Participant 4, Female

*“I still think it’s [wild meat] better than the livestock that’s mass-raised with hormones. You got chickens with three legs or something nowadays, because of the steroids and whatever they put in them.”*—Participant 12, Male

##### Medicinal and Healing Properties

Participants also emphasize the medicinal and healing properties of traditional foods, which can offer anti-inflammatory benefits, aid digestion, and support overall health:

*“…Some of them [wild food] help with anti-inflammatory, and you know, different kinds of foods that can be different kinds of things for you. It can help with like digestive problems, or it would be good for inflammation and things like that.”*—Participant 5, Female

##### Alignment with Indigenous Biology

The alignment of traditional foods with Indigenous biology is highlighted as an important aspect of their health benefits. These foods match the nutritional needs inherited from ancestors, supporting the belief in the natural compatibility of these foods with Indigenous peoples’ health:

*“From an indigenous perspective, our bodies were used to very lean meats, berries, etc. … Our bodies are meant to eat those foods, lean meats. But our sedentary lifestyle is not good either.”*—Participant 1, Female

#### 3.2.2. Theme 2: Cultural and Community Connection

This theme explores how traditional foods serve as vital links to cultural heritage, community cohesion, and the intergenerational transfer of knowledge. Participants shared how traditional foods not only nourish the body but also fortify connections to their identity, ancestors, and the land.

##### Cultural Heritage and Identity

Traditional foods are deeply intertwined with cultural heritage and identity, providing a tangible connection to ancestors and traditional ways of life:

*“You’re also fostering a sense of community because a lot of the times we feel so disconnected with the way we’re living now versus how we used to live when we were younger… it’s a way for us to stay connected as well, and gather still… It helps us maintain some of our traditions in our culture… So it’s more than just feeding your family, you know. It’s maintaining your identity as well.”*—Participant 4, Female

##### Community and Family Bonding

The preparation and consumption of traditional foods foster a sense of community and strengthen family bonds. Sharing food is seen as an act of healing, a way to fill up freezers with berries, and a method of providing for families:

*“That connection to home, to family, to the land, motivates me. And I guess, just that knowledge, knowing that that’s what my ancestors and my family and my community have always done.”*—Participant 11, Female

##### Intergenerational Knowledge Transfer

The preparation and sharing of traditional foods are crucial for the intergenerational transfer of knowledge. Through these practices, younger generations learn not only about food preparation but also about their cultural heritage and the significance of living in harmony with the environment:

*“While preparing traditional foods, you’re passing on that knowledge of how to make specific dried meats and what kind of wood you need to use and how to find that wood.”*—Participant 3, Female

#### 3.2.3. Theme 3: Physical and Mental Well-Being

The theme of physical and mental well-being highlights how traditional food practices contribute significantly to both physical health and emotional healing. Engaging in activities related to traditional foods fosters a deeper connection to nature, encourages physical activity, and serves as a source of stress relief and emotional healing.

##### Physical Activity and Connection to Nature

Participants shared how the processes involved in harvesting, preparing, and gathering traditional foods inherently encourage physical activity and foster a meaningful connection with the natural environment:

*“When you start to rely on traditional food, it means you’re moving around. Fiddlehead picking is a three-day process of washing them, cleaning them, picking them, and preparing them.”*—Participant 1, Female

##### Stress Relief and Emotional Healing

The preparation and consumption of traditional foods are deeply intertwined with emotional well-being, providing avenues for stress relief, emotional healing, and the strengthening of familial bonds:

*“And to be able to provide for families, that’s healing for us as well, to fill up the freezers full of berries.”*—Participant 1, Female

*“…when you go to the bush and you’re surrounded by the energy of the forest. That’s healing energy for us”*—Participant 1, Female

Furthermore, the engagement with traditional foods and the associated activities—such as hunting, gathering, and harvesting—provide a sense of purpose, accomplishment, and fulfillment, contributing to an overall sense of well-being:

*“Because I have a close connection with the land, so I feel obviously a sense of more connection with the animal when I’m eating it. It is more health-wise. It’s always just focused on mental health.”*—Participant 11, Female

#### 3.2.4. Theme 4: Economic and Cost Benefits

The theme of economic and cost benefits highlights how traditional food practices offer significant financial advantages and promote self-sufficiency within Indigenous communities.

##### Cost-Effectiveness and Self-Sufficiency

Participants emphasized the cost-effectiveness of relying on traditional food sources, which not only reduces grocery expenses but also fosters a sense of independence and sustainability:

*“When my family hunts, they try to fill our freezer, so that’ll last us a whole season. So it’s more cost effective”*—Participant 4, Female

*“For me the benefit for the wild meat is when I do get it, it’s given to me. So sometimes my reserve will do a hunt, and then it’s to share with all members of the band. And so that’s once a year that we get to reap the benefits of that. We just have to pay for the transport.”*—Participant 10, Female

##### Trade and Resource Sharing

The aspect of trade and resource sharing within traditional food practices is notably beneficial from an economic standpoint. This practice not only encapsulates the essence of community living but also presents a viable economic strategy for managing resources efficiently. By engaging in the exchange of goods, individuals and families can significantly reduce their food expenses, contributing to greater economic stability within the community:

*“But so my uncles go out and hunt and fish, and we always trade. We rely on family members and trade in. So every time a moose is killed, Mom and I go up there with our baking, or canning, or soup, or whatever we have. And we just trade.”*—Participant 1, Female

#### 3.2.5. Theme 5: Environmental and Ethical Considerations

Participants mentioned environmental and ethical considerations, emphasizing the profound respect Indigenous communities hold for sustainable practices and ethical sourcing of food. This theme explores how traditional food practices are intertwined with stewardship of the land, ethical treatment of animals, and the preservation of ecosystems, underscoring a holistic approach to health and well-being that extends beyond the individual to encompass the environment and future generations.

##### Sustainable and Ethical Sourcing

Participants highlighted the importance of ethically sourced foods, rooted in traditional protocols and a deep understanding of the natural world:

*“When you’re eating traditionally, you know that the food was ethically sourced because we follow protocols when we take from the land.”*—Participant 4, Female

The intimate knowledge of the sourcing and processing of traditional foods reflects a broader ethical stance towards consumption, emphasizing the importance of understanding and respecting where our food comes from:

*“Because I know exactly where it comes from, I know how it was like killed and all the process through it. So like knowing where it comes from, know exactly why. And I feel like the relationship too and I feel that adds to that sense of like health.”*—Participant 11, Female

##### Connection to the Land and Animals

This aspect of traditional food practices highlights a deep connection to the land and animals, highlighting the importance of maintaining a respectful and reciprocal relationship with nature:

*“For a lot of people, it [traditional food] connects them to the land they’re living on.”*—Participant 9, Male

*“The connection to the land and the animals, I feel like that’s such a big part of the reason.”*—Participant 11, Female

#### 3.2.6. Theme 6: Personal Preferences and Experiences

This theme sheds light on the unique personal preferences and experiences that underline the intrinsic value of traditional foods. From the distinctive tastes that connect individuals to their heritage to the nostalgic memories of family and cultural practices, this theme encapsulates the deep-rooted connections between traditional foods, personal history, and sensory satisfaction.

##### Taste

The distinctive taste of traditional foods is often highlighted as a primary factor for their preference. Participants express a profound appreciation for the natural and richer flavors found in traditional dishes, which are perceived as incomparable to commercially available options:

*“It’s pretty much like gold. If you can get a piece of like dried moose meat, it’s so delicious.”*—Participant 3, Female

*“Because it’s free, and I just grew up with it. I prefer the taste.”*—Participant 13, Male

##### Nostalgia and Personal History

The preparation and consumption of traditional foods often evoke strong feelings of nostalgia and a sense of continuity with past generations:

*“Nostalgia is a really big one. Just because my kokum (grandmother) is long past now, but she is kind of the one who taught me…”*—Participant 3, Female

*“That was my diet growing up on a reservation. My parents didn’t have to go to the supermarket. It was just whatever animal they brought home. So, that was our meal.”*—Participant 12, Male

**Figure 2:**
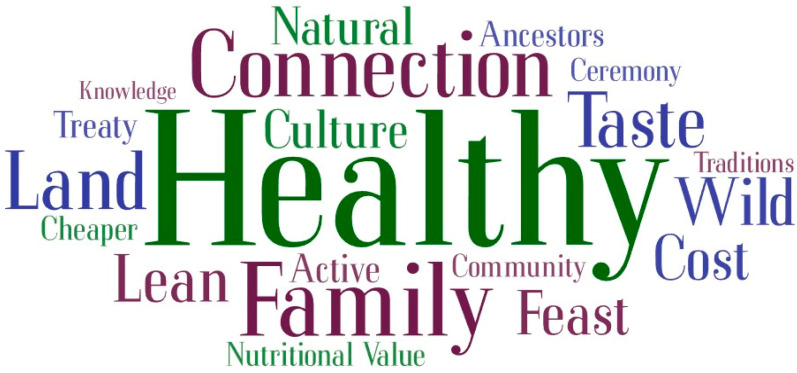
Word cloud of frequently used words in interviews on benefits of traditional foods.

Figure 2 displays a word cloud of the most frequently mentioned words from interviews discussing the benefits of traditional foods.

### 3.3. Risks of Traditional Foods

Using thematic analysis, three primary themes were identified that highlight the risks associated with traditional foods: (1) Disease Transmission and Health Risks from Wild Game; (2) Contamination and Pollution in Traditional Food Sources; and (3) Physical and Occupational Risks in Hunting and Gathering (Table 3).

#### 3.3.1. Theme 1: Disease Transmission and Health Risks from Wild Game

The consumption of traditional foods, particularly wild game, raises concerns regarding disease transmission and health risks. Participants express apprehensions about chronic wasting disease (CWD) and other health risks that could potentially arise from consuming affected animals.

Participants voiced specific worries about chronic wasting disease affecting deer and moose populations in Saskatchewan, highlighting the need for caution and awareness when consuming such game:

*“So moose meat, for example … the global chronic wasting disease…We know to stay away from that.…When I did the study in the eighties … I stopped eating game meat because of that. And just because of the fear of you know getting chronic wasting or Creutzfeldt–Jakob disease.”*—Participant 1, Female

The risk is not limited to larger game; smaller animals also pose health risks due to parasites and infections, emphasizing the importance of thorough inspection during processing:

*“Sometimes, with rabbits, they get an infection in their bones, and then there are little worms and stuff like that. So you have to be careful when you’re taking part in dismembering the animal, that you check for infections and stuff.”*—Participant 13, Male

#### 3.3.2. Theme 2: Contamination and Pollution in Traditional Food Sources

This theme addresses the growing concerns related to environmental pollution and its impact on the quality and safety of traditional foods. Participants highlight issues such as mercury content in fish, the influence of human activities on wildlife, and the repercussions of agricultural practices on the environment:

*“The reason why they [fish] are diseased is because of humans. They’re all polluted. They’re all full of gasoline and oil, and it’s disgusting. So that affects the fish that affects the moose feed that affects everything.”*—Participant 1, Female

*“I wouldn’t eat any of the deer around here because they eat garbage and stuff like that, same with other animals like bears. You wouldn’t really eat any of that unless you were in a far northern community where there was no garbage or anything, because they eat garbage, and then the meat gets gross.”*—Participant 13, Male

The increasing mercury content in fish is a significant concern, prompting a cautious approach to consuming fish from certain areas:

*“And like fish, that’s just been changing with higher mercury content. So it’s just things like that you have to watch out for.”*—Participant 13, Male

Participants also express worries about the indirect effects of agricultural practices, such as the use of pesticides and herbicides, on wildlife and consequently on the quality of traditional foods:

*“It’s about the pesticides that are out there that farmers are using. And so, whether or not the animals are consuming that or have any contact with that. That’d be my only concern with the quality of the traditional foods now. Like even the berries. The wind could blow the pesticides into the berry bushes that we consume, as well like Saskatoon berries.”*—Participant 12, Male

#### 3.3.3. Theme 3: Physical and Occupational Risks in Hunting and Gathering

This theme explores the inherent dangers associated with traditional methods of acquiring food. These risks, highlighted by the participants, range from accidents during hunting expeditions to environmental challenges that exacerbate the hazards involved.

Participants shared personal accounts and concerns about the potential for accidents while hunting, emphasizing the unpredictable nature of such activities:

*“My dad did get into a hunting accident 4 or 5 years ago… It was a freak accident. He shot himself by accident while he was out hunting in the field. So there’s those like occupational hazards, you never know.”*—Participant 4, Female

The changing climate and environmental conditions pose additional risks to those engaging in traditional hunting and gathering, affecting safety and the predictability of natural landscapes:

*“In the winter, I tend to worry a lot between winter and spring, when my dad and brothers go hunting because if you’re up in the north, it’s a lot of lakes up there and there’s been times where they go through the ice, or you don’t know like with the way the environment’s been changing, too…So yeah, the climate causes risks, too.”*—Participant 4, Female

**Figure 3:**
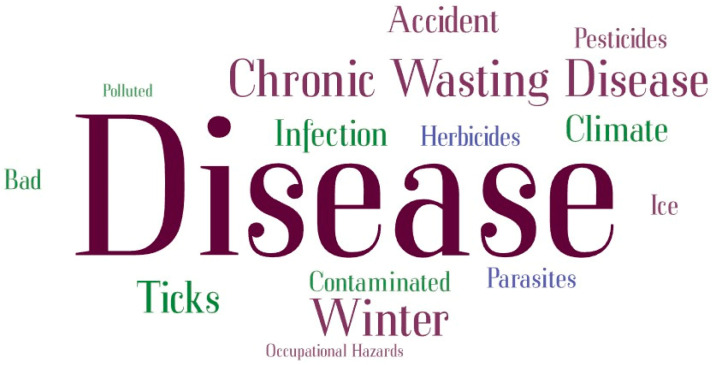
Word cloud of frequently used words in interviews on risks of traditional foods.

Figure 3 shows a word cloud of the most frequently mentioned words from interviews discussing the risks of traditional foods.

### 3.4. Barriers to the Consumption of Traditional Foods

Several key barriers to the consumption of traditional foods were identified and categorized into seven themes. These included (1) Economic and Financial Constraints; (2) Access and Availability Issues; (3) Cultural and Knowledge Gaps; (4) Regulatory and Policy Barriers; (5) Health and Safety Concerns; (6) Lifestyle and Practical Constraints; and (7) Environmental and Sustainability Concerns (Table 4).

#### 3.4.1. Theme 1: Economic and Financial Constraints

Economic and financial constraints emerge as significant barriers to the consumption of traditional foods among participants. The challenges of living under or close to the poverty line restrict access to nutrient-rich traditional foods, exacerbating health disparities.

##### Poverty and Financial Limitations

Participants highlight how poverty and financial limitations severely restrict their ability to access traditional foods, which are often perceived as more nutritious but less accessible due to economic constraints:

*“I think that people who are indigenous and who are urban live under that poverty line, and unfortunately they don’t have access to that type of nutrients [from traditional foods].”*—Participant 1, Female

##### Cost of Traditional Foods and Their Procurement

Participants identify the financial challenges associated with obtaining traditional foods, noting the high costs of hunting, gathering, and purchasing these foods compared to more accessible, albeit less nutritious, grocery store options:

*“It’ll be easier to go to the store to buy a pack of hamburger instead of buying a pack of hamburger that was made from a deer. It would cost a lot more for that piece of meat that’s been cut by the butcher.”*—Participant 5, Female

A common desire among urban Indigenous participants is for traditional foods to be more affordable and readily available. This would facilitate healthier eating habits and help preserve cultural practices:

*“If they if traditional foods were sold in the store and cheaper, I would definitely buy it.”*—Participant 10, Female

#### 3.4.2. Theme 2: Access and Availability Issues

Urban Indigenous participants highlight significant barriers to accessing traditional foods due to urbanization, limited hunting and gathering opportunities, dependency on social networks, and the impacts of seasonal variability on food availability.

##### Urbanization and Loss of Access

Participants note the challenges posed by urban living, where traditional methods of hunting and gathering are not feasible, leading to a reliance on alternative sources for traditional foods:

*“I know they used to have rabbits, duck, deer, moose, elk, and all those kinds of food but now it’s so rare. I know in the far North they still do rely on that but in the cities it’s very rare. You have to know somebody who’s a hunter in order to get them.”*—Participant 7, Female

##### Limited Hunting and Gathering Opportunities

Urban settings present unique obstacles to hunting and gathering, with farmland restrictions and the logistical difficulties of traveling to traditional territories:

*“Being in an urban setting, I guess, for my brothers, for example, harder to find places to hunt because there’s so much farmland… they’re not able to hunt freely up where we’re living.”*—Participant 4, Female

##### Reliance on Social Networks

Access to traditional foods often depends on connections within the community, highlighting a reliance on family and friends who hunt or gather:

*“There’s usually reliance on one male or a couple of males to provide those traditional foods and like in my family, the Patriarch, he passed away a few years ago, so I haven’t had moose meat in a long time.”*—Participant 3, Female

##### Limited Frequency and Occasion-Based Consumption

Participants describe traditional foods as primarily consumed during ceremonial events, indicating a gap in everyday access:

*“When there’s ceremonies I go on or when the First Nations gather, it’s called a feast, so they do like a ceremonial thing, and they pray for the food, and then they give everybody it. So that would be the only time things like would be accessible there.”*—Participant 5, Female

The necessity of being invited to these events further limits opportunities for consumption, highlighting an exclusivity based on community connections and specific occasions:

*“You have to be invited to these things [ceremonies]. So you can’t just go show up to one.”*—Participant 5, Female

##### Seasonal Variability

Seasonal changes affect the availability of traditional foods, with some participants noting a decline in natural resources and the specific times of year when hunting and gathering are more fruitful:

*“In the summer, we try to go to our fishing camp at least once a year, and the whole time we’re there we try to eat traditional foods the whole time, because it’s accessible there… So in the summer is definitely a good time at our camp. So seasonally, I would say it changes.”*—Participant 4, Female

##### Decreased Natural Availability and Accessibility

Environmental changes and a decrease in natural resources have led to reduced availability of traditional foods, impacting the diets of urban Indigenous families:

*“There used to be so much different kinds of choice for berries that you could eat here but now we can rarely find them unless somebody actually starts planting them on their own.”*—Participant 7, Female

#### 3.4.3. Theme 3: Cultural and Knowledge Gaps

Urban Indigenous participants identify significant barriers in the consumption of traditional foods related to cultural disconnection and knowledge gaps. These barriers arise from historical trauma, particularly the legacy of residential schools, and the challenges of transmitting traditional knowledge across generations within an urban context.

##### Impact of Residential Schools

The traumatic legacy of residential schools has disrupted the transmission of knowledge about traditional foods and practices:

*“There is an aspect of why my uncle didn’t pass on any of his information …because he was in a residential school, so he was very quiet and he never taught any of his kids how to hunt traditionally, or where to go.”*—Participant 3, Female

##### Loss of Generational Transfer of Knowledge

The interruption of knowledge transfer due to historical and familial disruptions results in gaps in skills and understanding necessary for engaging with traditional food practices:

*“There’s some things that I wouldn’t specifically go out and do because I was not taught… I don’t attend ceremonial feasts because I haven’t grown up with ceremonies a lot. So I don’t have that knowledge”*—Participant 11, Female

Participants express a desire to reconnect with or learn ancestral practices, acknowledging the barriers posed by urban living:

*“I’m trying to relearn some of the things I wasn’t able to learn because I’m in an urban setting like I’ve been here since I was 14 years old. So there’s a lot of cultural teachings I didn’t get.”*—Participant 4, Female

##### Cultural Disconnection and Lack of Exposure in Early Life

Participants express feelings of disconnection from their cultural roots and traditions, often attributed to urban living and lack of exposure during formative years:

*“When you move like from your home community to an urban setting, you lose that connection you have to your people… a lot of our norms and cultures and traditions it’s based around food and eating.”*—Participant 4, Female

#### 3.4.4. Theme 4: Regulatory and Policy Barriers

Urban Indigenous participants point out specific regulatory and policy barriers that significantly impede their access to and consumption of traditional foods. These barriers include strict food regulations that affect the serving and sale of traditional meats, as well as conservation policies that restrict hunting practices.

##### Food Regulations and Restrictions

Strict Canadian food regulations present considerable challenges in serving and selling traditional meats within formal establishments. This regulatory environment limits the availability of traditional foods in urban areas:

*“I used to serve traditional foods at the restaurant that I used to run… and there were certain things, of course, that you can’t serve because Canadian food regulations do not allow it. They don’t allow for me to sell moose meat or serve moose meat. So I wasn’t able to do that.”*—Participant 1, Female

##### Conservation Policies and Hunting Rights

Conservation policies and the complexities of hunting rights, especially when involving Indigenous and non-Indigenous family members, further complicate access to traditional foods. These policies can inadvertently foster racial divisions and negatively impact food security for Indigenous families:

*“There’s a lot of policies that cause barriers too like conservation policies. For example, I’m Indigenous, but my spouse is non-Indigenous… So he’s not able to hunt with my family because there’s policies that cause a barrier. Yes, my brothers have treaty rights, and they have the right to hunt and provide. But once my spouse joins them, I’m not sure clearly about the policy, but it makes it so that they kind of lose their right to hunt when he’s there.”*—Participant 4, Female

#### 3.4.5. Theme 5: Health and Safety Concerns

Urban Indigenous participants have raised significant health and safety concerns regarding the consumption of traditional foods, particularly related to contamination and disease. These concerns stem from environmental pollution and the potential for disease transmission from wildlife to humans, influencing their decisions about consuming traditional foods.

Reports of diseased fish and concerns about contamination have made participants wary of consuming traditional foods sourced from their local environments:

*“Recent thing that’s been witnessed are diseased fish…some of them will be full of parasites. So we don’t eat stuff like that.”*—Participant 1, Female

*“I wouldn’t even eat the fish because the sewer goes into the river, and everything from the streets. So, I wouldn’t eat any of the wild animals around here.”*—Participant 13, Male

The increasing presence of contaminants in the environment has led to a reevaluation of the healthiness of traditional foods:

*“I would like to say that they are healthier, since they are not processed but that was years ago and now I am not so sure. […] But right now everything is so contaminated.”*—Participant 7, Female

#### 3.4.6. Theme 6: Lifestyle and Practical Constraints

Urban Indigenous participants highlighted several lifestyle and practical constraints that impede their ability to consume traditional foods. These barriers include time and effort required for preparation, storage and preservation challenges, and lifestyle and dietary shifts, reflecting the complexity of integrating traditional foods into contemporary urban living.

##### Time and Effort for Preparation

Participants pointed out that the convenience of store-bought foods often outweighs the time-consuming processes involved in preparing traditional foods:

*“It’s basically the transport and the time. And then it costs money to have it shipped. So if I want to get buffalo ground beef from my reserve or any kind of wild meat, I actually have to kind of go in, it has to be butchered, and so it’s a process. It’s just not readily on the shelf, and then it has to come back into the city”*—Participant 10, Female

*“Having the time, being busy, that’s one of the reasons why we don’t do a lot of our traditional things—being busy nowadays.”*—Participant 14, Male

##### Storage and Preservation Challenges

Concerns about the storage and preservation of traditional foods, especially given their perishability, were also mentioned as significant barriers:

*“Then, there’s the challenge of where to store everything. Because, as mentioned, produce can go bad quickly.”*—Participant 8, Female

##### Lifestyle and Dietary Shifts

Changes in dietary preferences and the influence of colonization on Indigenous foodways were identified as factors contributing to the decreased consumption of traditional foods:

*“When colonization came and the Europeans brought in different types of food, it like kind of messed up with their digestive system, their health system and immune system, because it introduced different sugars and all these other things that their bodies wasn’t used to.”*—Participant 11, Female

#### 3.4.7. Theme 7: Environmental and Sustainability Concerns

Urban Indigenous participants express significant sustainability and scarcity concerns as barriers to the consumption of traditional foods. These concerns highlight the direct impact of environmental changes and conservation issues on the availability of traditional food sources, emphasizing the need for sustainable practices to ensure the continuation of these foodways for future generations.

##### Sustainability and Scarcity Concerns

Participants voiced worries about overhunting, environmental degradation, and the sustainability of wildlife populations, underscoring a respectful and conscientious approach to consuming traditional foods:

*“If there was conservation issues like, if we were being told this population of animals being wiped out then you have to respect that and because you wanna think about future generations to not only ourselves.”*—Participant 4, Female

##### Impact of Climate Change on Traditional Foods

The effects of climate change on traditional food sources, such as altered animal behavior, reduced availability of plants and animals, and extreme weather conditions, were also highlighted as significant barriers:

*“If we have a really warm winter, you’re gonna have massive amounts of deer ticks and moose ticks.”*—Participant 1, Female

The changing climate also directly affects plant-based food sources:

*“What happened was, come May, we didn’t get enough rain, so the fiddleheads didn’t come up right away and then around May 28th, there was a frost, and the fiddleheads that did come up all froze. We were devastated.”*—Participant 1, Female

### 3.5. Facilitators for the Consumption of Traditional Foods

Several key facilitators for the consumption of traditional foods were identified and categorized into four themes: (1) Family and Community Networks; (2) Proactive Engagement in Traditional Practices; (3) Transmission of Cultural Knowledge and Practices; and (4) Cultural and Ceremonial Gatherings.

#### 3.5.1. Theme 1: Family and Community Networks

Family and community networks play a crucial role in facilitating the consumption of traditional foods among urban Indigenous peoples. These networks provide essential links to traditional practices and access to foods that might otherwise be inaccessible due to urban living conditions. Many urban Indigenous individuals rely on family members who live in or near traditional lands to supply them with traditional foods:

*“Sometimes my brother goes up north and he’ll bring back like a piece of frozen moose meat, or like a chunk of dry meat.”*—Participant 3, Female

Traditional foods are often shared among family members, extending the reach of these foods beyond immediate geographical limitations:

*“I get my fish and my moose meat from my uncle, and Mom and I go pick our berries every year”*—Participant 1, Female

#### 3.5.2. Theme 2: Proactive Engagement in Traditional Practices

Proactive engagement in traditional practices is a significant facilitator for the consumption of traditional foods among urban Indigenous peoples. Individuals take initiative to organize trips and activities that allow them to procure traditional foods directly from the source, ensuring that they have access to these culturally important foods regardless of their urban setting.

*“I’m planning a hunting trip next weekend. So I’m going to go hunting. So we’ll have some more wild meat in our fridge.”*—Participant 14, Male

#### 3.5.3. Theme 3: Transmission of Cultural Knowledge and Practices

The transmission of cultural knowledge and practices is a pivotal facilitator for the consumption of traditional foods among urban Indigenous peoples. This transmission ensures that traditional skills and values are passed down through generations, allowing urban Indigenous communities to maintain their cultural heritage and food practices despite the urban environment.

Educators and community leaders play crucial roles in conveying knowledge about traditional food procurement and preparation. This education is often formalized through community programs or conveyed informally through family teachings.

*“…that’s the type of programs that need to take place on First Nations because they teach about the values of foraging, hunting, fishing, and growing their gardens.”*—Participant 1, Female

*“We are very fortunate to be able to go out and hunt and provide for our families, and cook our own home meals, and provide that healthy living that some people can’t afford… we try to raise our kids that way.”*—Participant 14, Male

#### 3.5.4. Theme 4: Cultural and Ceremonial Gatherings

Cultural and ceremonial gatherings play a significant role in facilitating the consumption of traditional foods among urban Indigenous peoples. Traditional foods are often central to various cultural ceremonies, including feasts, sweats, and other significant community events. These occasions ensure that traditional foods are not only preserved but celebrated within the community.

*“For me, personally, I usually consume them when I attend a feast or when I go to a sweat lodge.”*—Participant 8, Female

Life events such as funerals, weddings, and community engagement meetings on the reserve frequently include traditional foods, linking everyday life with cultural heritage.

*“If there’s a funeral, a wedding, or any kind of engagement with my reserve in particular, if they have community engagement meetings, they have traditional foods.”*—Participant 8, Female

## 4. Discussion

**Figure 4:**
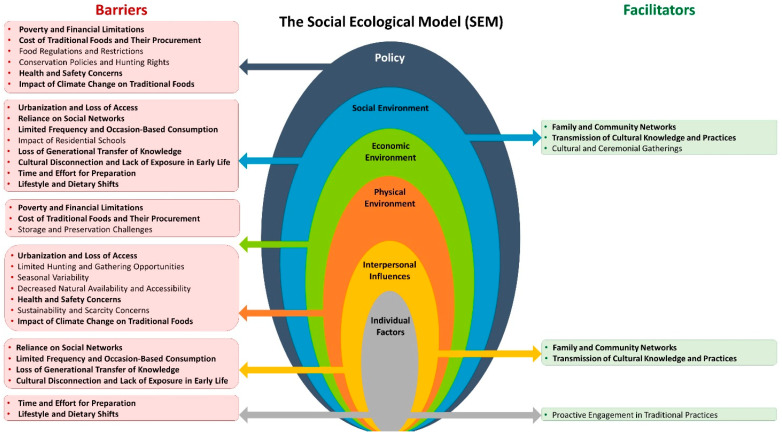
Conceptual framework of barriers and facilitators to consumption of traditional foods among urban Indigenous populations based on the Social Ecological Model (SEM). The bolded items are those that are repeated across multiple layers.

In this study, we explored for the first time the complex interplay between cultural heritage, health practices, and urban living among Indigenous communities in Saskatchewan in relation to traditional foods. Our findings underscore the profound connection between traditional diets and Indigenous identity, with participants expressing a strong sense of cultural continuity and community cohesion facilitated through dietary practices. Notably, the health benefits associated with traditional foods—such as nutrient density and lack of industrial processing—were frequently highlighted, reflecting a deep-rooted belief in the intrinsic value of these foods for physical and cultural well-being. However, alongside these perceived benefits, participants also expressed significant concerns related to accessibility, sustainability, and safety of traditional foods, which are compounded by urban living conditions and modern environmental challenges. Our study results have led to the creation of a conceptual framework, enriched with an intersectionality lens (Figure 4), based on the SEM. This framework addresses various levels of the SEM, including individual factors, interpersonal influences, physical environment, economic environment, social environment, and policy. Importantly, this framework guided the interpretation of our findings and was instrumental in developing the discussion section of our study.

The findings from our interviews with urban Indigenous peoples in Saskatchewan resonate with broader research highlighting the nutritional superiority and health benefits of traditional foods. Studies consistently report that traditional foods are integral to physical health due to their high nutrient contents and absence of industrial additives. Kuhnlein and Receveur (2007) noted that traditional animal and plant foods consumed in Canadian Arctic Indigenous diets have been increasingly displaced by market foods. Despite this shift, traditional foods continued to contribute a significant portion of daily energy and were shown to enhance the intake of essential nutrients such as protein, vitamins, and minerals when included even in small amounts [30]. Sheehy et al. (2014) further support these findings in their assessment of dietary adequacy among the Inuvialuit. Their results showed that those who consumed more traditional foods had significantly better nutrient profiles, including higher levels of protein, vitamins, and omega-3 fatty acids, and lower intakes of non-nutrient-dense foods [31]. Similarly, in another study conducted in Inuit populations, Sheehy et al. (2015) observed that individuals who consumed more traditional foods achieved better dietary adequacy and consumed diets richer in nutrients compared to their non-traditional counterparts [32]. Blanchet et al. (2020) compared health indicators and diet quality among Syilx Okanagan adults and found that those consuming traditional foods had significantly higher intakes of several key nutrients and better overall diet quality as measured by the Healthy Eating Index. Notably, traditional food consumers also consumed fewer ultra-processed products, illustrating how traditional diets can steer populations away from modern dietary pitfalls that contribute to chronic diseases [33]. Further, Gagné et al. (2012) provided insights into the dietary impacts of traditional foods on children in Nunavik, demonstrating that even minimal consumption of traditional foods like caribou and Arctic char significantly boosted nutrient intakes among children. Despite the low frequency of traditional food consumption, it resulted in markedly improved nutritional quality of children’s diets, reinforcing the potential benefits of integrating these foods into daily eating habits [7]. Together, these studies highlight the crucial role of traditional foods in sustaining the physical health of Indigenous populations through superior nutrition.

The cultural and community aspects of traditional foods highlighted in our study are strongly supported by existing literature, which underline the profound role these foods play in maintaining cultural heritage, fostering community bonds, and facilitating the intergenerational transfer of knowledge. Lambden et al. (2007) demonstrated that traditional foods are not only regarded as healthy and nutritious but also as essential elements of Arctic food security, contributing to physical fitness, health, and cultural continuity [34]. This aligns with our findings, where participants emphasized that traditional foods foster a sense of community, maintain cultural traditions, and provide a tangible link to their ancestry and identity. Robin and Cidro highlighted the importance of integrating traditional food practices in educational settings, noting that such programs strengthen Indigenous culture, identity, and relationships [35]. This mirrors the experiences of our participants, who stressed the importance of passing on knowledge about food preparation and cultural practices to younger generations [35]. The study by Snook et al. (2020) on the intricate relationship between Inuit communities and the Mealy Mountain Caribou emphasized not only the nutritional importance of caribou but also its profound significance in sustaining cultural practices and identities [36]. The research revealed that the caribou hunting practices, deeply rooted in the cultural fabric of the Inuit communities of Rigolet, Nunatsiavut, served multiple functions: they were vital for physical sustenance, cultural education, and social cohesion. Through 21 in-depth interviews and community open houses, Snook and colleagues uncovered that caribou hunting was not merely an activity for food provision but a complex cultural practice that involved significant emotional and social dimensions [36]. Participants described caribou hunting as a source of pride, excitement, and joy, contributing to a strong sense of identity and community well-being. This resonates with our findings, where participants highlighted the stress-relieving and healing aspects of engaging in traditional food activities, such as harvesting and preparing food, which provide a sense of purpose and fulfillment. However, at the policy level, food regulations, restrictions, and conservation policies, including hunting bans, limit access to traditional foods. For instance, Snook et al. (2020) pointed out that the disruptions caused by external regulations, such as hunting bans, have not only affected the availability of caribou but also the transmission of this critical cultural knowledge. Participants also expressed concerns over the erosion of cultural practices and the loss of traditional knowledge, particularly as older generations pass away [36]. Similarly, Newell et al. (2020) highlighted the importance of traditional food harvesting and sharing in maintaining cultural continuity and community health in Arctic Inuit communities [37]. The study described how activities like harvesting country food act as a “refresher for our mind, body, and soul”, offering both physical exercise and a mental health boost. Participants emphasized that spending time on the land, whether for harvesting or simply enjoying the natural environment, profoundly impacts individual health and community well-being [37]. This study supports our findings that engaging in traditional food practices fosters community cohesion, supports mental health, and facilitates the transmission of crucial cultural knowledge, which is especially vital in the face of external pressures like climate change, modernization, and urbanization. Tsuji et al. (2020) discussed the importance of on-the-land programs for the transfer of Indigenous knowledge, noting that such initiatives facilitate vertical transmission of cultural practices and enhance youth engagement with traditional activities [38]. Moreover, the authors highlighted social aspects such as collaborative efforts and interactions among community members who typically do not associate with each other, highlighting another dimension of community building through shared activities. Collectively, these studies reinforce the critical role of traditional foods in sustaining cultural heritage, community cohesion, and intergenerational knowledge transfer. These roles are particularly crucial in urban environments, where traditional practices are often challenged by the dynamics of modern life and the barriers identified across multiple levels of the SEM.

The economic and cost benefits of traditional food practices, as highlighted in our interviews, align closely with findings from the literature on sharing networks within Indigenous communities. We have previously demonstrated that our participants consider economic constraints and poverty to be major barriers to healthy eating [39]. Participants emphasized the cost-effectiveness and sustainability of traditional food sources, noting that hunting and sharing practices reduce grocery expenses and foster economic independence. This is supported by several studies that explore the economic dimensions of traditional food sharing at the economic-environment and social-environment levels of the SEM. Beaumier and Ford (2015) detailed how the availability of country food in Inuit households is largely dependent on the presence of full-time hunters, who ensure a steady supply of traditional foods like caribou. This study highlighted the vulnerability of households lacking a hunter, often relying on others for shared meat, which parallels our participants’ reliance on community networks for traditional food resources. The notion of ‘free’ country food, prevalent in Beaumier and Ford’s findings, resonates with our participants’ experiences of receiving shared food without direct costs, emphasizing the economic relief this practice provides to those with limited financial resources [40]. Gilbert et al. (2020) examined food sharing norms and their impact on community food security, noting that successful hunts often lead to the distribution of meat within and between communities, facilitated through various communication tools like local radio and social media [41]. This mirrors our findings where participants described trading and sharing traditional foods, such as moose meat and berries, among family members and community members, reinforcing economic stability and social bonds. Snook et al. (2020) further elucidated the importance of caribou in Inuit culture, livelihood, and food security, underscoring that the sharing of caribou meat is a key cultural practice. Participants in this study emphasized that beyond the nutritional value, the process of hunting and sharing caribou meat is integral to community cohesion and economic resilience [36]. Newell et al. (2020) supported these observations by demonstrating that harvesting and sharing country food are critical for linking food security, cultural continuity, and community health in Chesterfield Inlet. Their study emphasized that sharing country food is a traditional practice that supports not only food security but also financial stability within the community, resonating with our participants’ experiences of sharing food to reduce living costs and support each other financially [37]. Tsuji et al. (2020) also described the Sharing-the-Harvest programs, which facilitate the distribution of harvested geese within Cree communities, highlighting both vertical and horizontal knowledge transfer and the importance of sharing food from a food security perspective [38]. Together, these studies highlight the economic advantages and community resilience fostered by traditional food sharing networks. Our findings, consistent with the literature, highlight the significance of these practices in reducing costs, promoting self-sufficiency, and supporting community cohesion.

Our study prominently highlighted the impact of climate change on traditional food practices and the associated physical and occupational risks, echoing concerns raised in the literature about how environmental changes are affecting Indigenous food systems. Our participants expressed worries about the unpredictability and dangers of hunting and gathering due to shifting climate conditions, which have also been documented in several studies exploring the broader implications of climate change on traditional food accessibility and safety. These concerns span multiple levels of the SEM. At the policy level, there is a lack of comprehensive strategies to mitigate climate change impacts on traditional food systems. At the physical-environment level, climate-induced changes such as thinner ice and unpredictable weather patterns increase the risks associated with traditional food procurement practices. We have previously shown that 38.9% of urban Indigenous respondents in Saskatchewan identified climate change as a significant factor affecting traditional food harvesting and consumption [42]. Ford and Beaumier (2011) documented changes in environmental conditions in Igloolik, Nunavut, which have increased the danger of hunting and constrained access to hunting areas. These changes have led to acute shortages of traditional foods, mirroring our participants’ experiences with climate-induced challenges such as thinner ice and unpredictable weather patterns, which increase the risks associated with traditional food procurement practices [43]. Beaumier and Ford (2015) explored how climate-related changes, such as declining sea ice and altered animal migration patterns, have affected the food security of Inuit women in Arviat. These changes have created conditions where traditional food sources are less accessible, leading to increased reliance on less nutritious store-bought foods [40]. Guyot et al. (2006) reported on the observed changes in species, water levels, weather, and ice conditions in two northern Indigenous communities. Participants noted new species appearances, altered migration patterns, and fluctuating water levels, which have affected traditional harvesting practices [44]. Pufall et al. (2011) highlighted the concerns of Inuit residents in Nain, Nunatsiavut, regarding climate change’s impact on country foods, with worries about changing animal populations and the potential disappearance of key species [45]. Our participants similarly expressed concerns about how climate change might lead to the scarcity of traditional foods, exacerbating the risks and uncertainties of traditional hunting and gathering. Further, using data from First Nations Food, Nutrition, and Environment Study (FNFNES), Batal et al. (2021) found that climate change was frequently cited as negatively affecting the availability and accessibility of traditional foods across various ecozones in Canada. The study noted that these impacts were more pronounced in certain regions, affecting both the amount of traditional food available and the ability to access these foods [14]. Overall, the literature reinforces our findings on the significant impact of climate change on traditional food practices, emphasizing the increased risks and reduced availability of traditional foods. These challenges highlight the need for sustainable practices and adaptive strategies at multiple levels of the SEM to ensure the continuation and safety of traditional foodways for future generations.

In examining the various barriers to the consumption of traditional foods among urban Indigenous peoples in Saskatchewan, a complex interplay of economic, cultural, regulatory, and environmental factors emerges. These barriers, spanning multiple levels of the SEM (Figure 4), not only limit access but also affect the preservation of cultural identity and health equity within these communities. At the economic-environment level, our participants frequently cited economic barriers as a primary obstacle to accessing traditional foods. High costs associated with hunting, gathering, and purchasing traditional foods make them less accessible compared to store-bought alternatives, which are often less nutritious. This aligns with findings from our 2023 survey, where a notable percentage of respondents indicated that the expense of acquiring necessary equipment like bullets and fishing gear is prohibitive [42]. Additionally, Keith et al. (2018) highlighted similar themes, noting that the financial burden of healthy foods and the logistical challenges of food preparation due to busy schedules further complicate access to nutritious options [46]. At the physical-environment level, urbanization significantly restricts opportunities for hunting and gathering. Our participants and studies such as Skinner et al. (2013) identify environmental changes and high costs of hunting as barriers to traditional food acquisition. Urbanization also leads to a reliance on social networks for access to traditional foods, which underscores a vulnerability to food insecurity when those networks are unavailable or disrupted [47]. At the social-environment level, the legacy of residential schools has notably disrupted the transmission of traditional knowledge, leading to significant gaps in skills and cultural disconnection. This is particularly poignant in urban settings, where opportunities to engage with traditional practices are limited. Elliott et al. (2012) discussed the importance of incorporating Aboriginal perspectives in addressing these gaps, emphasizing that traditional knowledge is often sidelined in mainstream food security discussions [48]. At the policy level, strict food regulations and conservation policies create substantial barriers to accessing traditional foods. Our participants noted that regulations prevent the serving and sale of traditional meats and complicate hunting rights. Furthermore, Elliott et al. (2012) highlighted how government policies, such as those by the Department of Fisheries and Oceans, restrict Indigenous fishing rights off their territories, exacerbating access issues for those living off reserve [48]. Environmental concerns also play a significant role. Contamination and health concerns related to environmental pollution have made many urban Indigenous peoples wary of consuming locally sourced traditional foods. These concerns are reflected in the broader literature, which recognizes environmental pollutants as a factor reducing the availability of safe and healthy traditional foods [2,49]. At the individual level, the practical difficulties of integrating traditional food practices into modern urban lifestyles, such as the time required for preparation and the challenges of storage and preservation, were frequently mentioned by our participants. These barriers are echoed in Chan et al. (2006), who suggest that changes in lifestyle and cultural practices have led to decreased consumption of traditional foods [50]. In light of these findings, it is clear that addressing the barriers to traditional food consumption requires a multifaceted approach. This approach should consider economic support, the enhancement of cultural and food-related education, improvements in policy and regulatory frameworks to better support traditional practices, and the strengthening of community networks to enhance food security among urban Indigenous populations.

Implications: The implications of this research study are extensive and offer several pathways for enhancing public health, cultural preservation, and policy-making to support urban Indigenous communities in Saskatchewan. The study highlights the importance of traditional foods not only for nutritional health but also for maintaining cultural identity and fostering community cohesion. Consequently, policies are clearly needed to improve access to these foods and facilitate their integration into urban lifestyles. These implications can be addressed at multiple levels of the SEM. At the policy level, reducing regulatory barriers restricting the hunting, gathering, and distribution of traditional foods is essential. Policies should support Indigenous hunting and fishing rights and ensure that regulations do not impede the availability of traditional foods. Additionally, urban planning and public health policies should create supportive environments for traditional food practices, such as the development of culturally specific food markets or designated areas for community gatherings. At the interpersonal and social-environment levels, one pivotal area is the renewal of family and community relationships through initiatives that connect youth with Elders, thereby facilitating the intergenerational transfer of traditional knowledge [48]. This connection is vital for preserving cultural heritage and enhancing the social fabric of Indigenous communities. Encouraging the sharing and trade of traditional foods among community members can strengthen social bonds and improve food security. Food-related community programs that incorporate these elements can play a significant role in achieving these goals. These programs could include community gardens that grow traditional plants, workshops on traditional food preparation, and cultural festivals that celebrate Indigenous culinary traditions [51]. At the economic-environment level, programs that increase access to land and sea for traditional food gathering are crucial, as they enable Indigenous communities to practice their cultural food harvesting traditions, thereby promoting sustainability and self-reliance. These initiatives can reduce economic barriers by providing resources and support for traditional food practices, such as subsidies for hunting and fishing equipment or financial incentives for community-led food projects. At the physical-environment level, creating designated areas for community gardens and traditional food harvesting within urban settings can provide accessible spaces for practicing and celebrating Indigenous food traditions. Finally, at the individual and interpersonal levels, education initiatives focused on the nutritional benefits and preparation of traditional foods could empower younger generations and foster a deeper appreciation for their cultural heritage. Workshops and school-based programs that educate Indigenous youth about the nutritional, cultural, and environmental aspects of their food can strengthen cultural identity and promote healthy eating habits.

Strengths and limitations: In this study, we employed a qualitative approach to explore the perceptions and experiences of urban Indigenous individuals in Saskatchewan regarding traditional foods. The research was rigorously designed to ensure a deep and ethically sound exploration, employing inductive thematic analysis as recommended by the Consolidated Criteria for Reporting Qualitative Research (COREQ). Participants were recruited from Saskatoon, Regina, and Prince Albert, through collaborative efforts with local Indigenous organizations. This strategy enabled us to engage a diverse group of individuals who brought a wealth of perspectives to our study. The use of purposive and snowball sampling techniques ensured that our participant pool was not only relevant but also rich in experiences pertinent to our research aims. Our analysis was aided by NVivo, which supported a systematic examination of the transcripts. This process was further validated through participant verification, where individuals reviewed their interview transcripts to confirm accuracy, thus enhancing the credibility of our findings. Additionally, applying the SEM as a theoretical framework allowed for a comprehensive examination of the multiple layers of influence on traditional food practices, spanning from individual factors to broader policy environments. Despite these strengths, our study is not without limitations. The geographic focus was confined to urban centers within Saskatchewan, which may limit the applicability of our findings to other urban settings. The gender distribution of participants skewed towards females, with 10 females and 4 males participating in our interviews. This distribution potentially influenced the thematic outcomes and limited our ability to conduct gender-specific analyses, as recruiting male participants was challenging. Recruitment through specific community organizations could also impose a selection bias, as participants associated with these networks might not fully represent the broader urban Indigenous population. Furthermore, although the study sought to encompass a wide range of experiences, it may have missed essential viewpoints from participants who have limited access to technology or are less likely to engage in research studies.

## 5. Conclusions

This study identifies the pivotal role traditional foods play in the lives of urban Indigenous peoples in Saskatchewan, highlighting their profound impact on cultural identity, community cohesion, and health. Traditional foods are not merely sustenance but are deeply embedded in the cultural fabric, offering significant health benefits, strengthening familial and community bonds, and serving as a bridge to cultural heritage and ancestral practices. Despite the recognized benefits, our findings also highlight several substantial barriers that hinder the consistent consumption and accessibility of traditional foods. At the economic-environment level, barriers such as high costs associated with hunting, gathering, and purchasing traditional foods complicate efforts to maintain these foodways in urban settings. Regulatory and policy limitations at the policy level further restrict access, while environmental and sustainability concerns at the physical-environment level pose significant challenges. Additionally, the social environment is impacted by the loss of traditional knowledge and the challenges posed by urbanization, which threaten the continuity of these practices. To effectively support urban Indigenous populations, it is essential for policymakers, community leaders, and health practitioners to consider these findings and work collaboratively towards solutions that are culturally appropriate, build on community strengths, and respond to community-identified needs. By fostering environments that support the availability and accessibility of traditional foods at multiple levels of the SEM, we can contribute to the health, well-being, and cultural vitality of urban Indigenous peoples.

Moving forward, it is crucial that subsequent research builds upon these findings to deepen our understanding and address the emergent challenges. Future studies should explore the impact of urbanization on traditional food practices across different cities and provinces to identify common patterns or unique challenges. Longitudinal research would also provide valuable insights into the evolution of these practices and the long-term effectiveness of policies aimed at enhancing traditional food access. We encourage future researchers to adopt interdisciplinary approaches that integrate cultural, nutritional, and policy analyses to provide comprehensive solutions to support and revitalize traditional food practices among urban Indigenous communities.

## Figures and Tables

**Table 1:** Participants’ characteristics.

	*n* (%)
Indigenous Identity	
First Nation	12 (85.8%)
Métis	1 (7.1%)
Unknown	1 (7.1%)
Place of Residence	
Saskatoon	7 (50.0%)
Regina	5 (35.7%)
Prince Albert	2 (14.3%)
Gender	
Female	10 (71.4%)
Male	4 (28.6%)
Education Level	
High School Education	3 (21.4%)
Undergraduate Education	8 (57.1%)
Graduate Education	1 (7.1%)
Non-degree Certification	1 (7.1%)
Professional Certification	1 (7.1%)
Employment Status	
Employed	8 (57.1%)
Unemployed	6 (42.9%)
Household Size	
1	2 (14.3%)
2	4 (28.6%)
3	2 (14.3%)
4	3 (21.4%)
≥5	3 (21.4%)
Home Ownership	
Own	2 (14.3%)
Rent	11 (78.6%)
Unknown	1 (7.1%)
	**Mean ± SD (Range)**
Age (Years)	39.8 ± 11.7 (21–61)
Annual Household Income (CAD)	CAD $72,600 ± CAD $30,692 (CAD $20,000–CAD $120,000)

**Table 2:** Themes and subthemes for benefits of traditional foods.

Theme #	Themes	Subthemes
Theme 1	Health and Nutritional Benefits	Nutrient Density and Quality
		Lack of Industrial Processing and Additives
		Medicinal and Healing Properties
		Alignment with Indigenous Biology
Theme 2	Cultural and Community Connection	Cultural Heritage and Identity
		Community and Family Bonding
		Intergenerational Knowledge Transfer
Theme 3	Physical and Mental Well-being	Physical Activity and Connection to Nature
		Stress Relief and Emotional Healing
Theme 4	Economic and Cost Benefits	Cost-Effectiveness and Self-Sufficiency
		Trade and Resource Sharing
Theme 5	Environmental and Ethical Considerations	Sustainable and Ethical Sourcing
		Connection to the Land and Animals
Theme 6	Personal Preferences and Experiences	Taste
		Nostalgia and Personal History

**Table 3:** Themes for risks of traditional foods.

Theme #	Themes
Theme 1	Disease Transmission and Health Risks from Wild Game
Theme 2	Contamination and Pollution in Traditional Food Sources
Theme 3	Physical and Occupational Risks in Hunting and Gathering

**Table 4:** Themes and subthemes for barriers to the consumption of traditional foods.

Theme #	Themes	Subthemes
Theme 1	Economic and Financial Constraints	Poverty and Financial Limitations
		Cost of Traditional Foods and Their Procurement
Theme 2	Access and Availability Issues	Urbanization and Loss of Access
		Limited Hunting and Gathering Opportunities
		Reliance on Social Networks
		Limited Frequency and Occasion-Based Consumption
		Seasonal Variability
		Decreased Natural Availability and Accessibility
Theme 3	Cultural and Knowledge Gaps	Impact of Residential Schools
		Loss of Generational Transfer of Knowledge
		Cultural Disconnection and Lack of Exposure in Early Life
Theme 4	Regulatory and Policy Barriers	Food Regulations and Restrictions
		Conservation Policies and Hunting Rights
Theme 5	Health and Safety Concerns	
Theme 6	Lifestyle and Practical Constraints	Time and Effort for Preparation
		Storage and Preservation Challenges
		Lifestyle and Dietary Shifts
Theme 7	Environmental and Sustainability Concerns	Sustainability and Scarcity Concerns
		Impact of Climate Change on Traditional Foods

## Data Availability

The data presented in this study are available on request from the corresponding author. The data are not publicly available due to privacy and ethical considerations related to working with Indigenous communities.
